# Vitamin D and Secondary Hyperparathyroidism in Chronic Kidney Disease: A Critical Appraisal of the Past, Present, and the Future

**DOI:** 10.3390/nu14153009

**Published:** 2022-07-22

**Authors:** Vincent Brandenburg, Markus Ketteler

**Affiliations:** 1 Department of Cardiology and Nephrology, Rhein-Maas-Klinikum Würselen, Mauerfeldchen 25, 52146 Würselen, Germany; 2 Departmentof General Internal Medicine and Nephrology, Robert-Bosch Hospital, Auerbachstraße 110, 70376 Stuttgart, Germany; Markus.Ketteler@rbk.de

**Keywords:** vitamin D, vitamin D insufficiency, secondary hyperparathyroidism, parathyroid hormone, chronic kidney disease, chronic kidney disease–mineral and bone disorder

## Abstract

The association between vitamin D deficiency and especially critical shortage of active vitamin D (1,25-dihydroxyvitamin D, calcitriol) with the development of secondary hyperparathyroidism (sHPT) is a well-known fact in patients with chronic kidney disease (CKD). The association between sHPT and important clinical outcomes, such as kidney disease progression, fractures, cardiovascular events, and mortality, has turned the prevention and the control of HPT into a core issue of patients with CKD and on dialysis. However, vitamin D therapy entails the risk of unwanted side effects, such as hypercalcemia and hyperphosphatemia. This review summarizes the developments of vitamin D therapies in CKD patients of the last decades, from calcitriol substitution to extended-release calcifediol. In view of the study situation for vitamin D insufficiency and sHPT in CKD patients, we conclude that the nephrology community has to solve three core issues: (1) What is the optimal parathyroid hormone (PTH) target level for CKD and dialysis patients? (2) What is the optimal vitamin D level to support optimal PTH titration? (3) How can sHPT treatment support reduction in the occurrence of hard renal and cardiovascular events in CKD and dialysis patients?

## 1. Introduction

### 1.1. Aetiology and Prevalence of Vitamin D in Chronic Kidney Disease

With over a billion cases, vitamin D deficiency has become a major burden on public health [1]. One of the main causes for vitamin D deficiency is chronic kidney disease (CKD) [1]. Chronic kidney disease is defined as the progressive loss of kidney function that persists for at least 3 months, is irreversible, and leads ultimately to end-stage renal disease (ESRD) [2,3]. Declining kidney function can be determined by the estimated glomerular filtration rate (eGFR; ranges from 120 to 0 mL/min/1.73 m²) or the corresponding CKD stage (ranges from G1 to G5), with lower eGFR-values indicating higher CKD stages: an eGFR persistently below 60 mL/min/1.73 m² (stage G3a) indicates CKD. As studies have shown, the progressive decline of the eGFR is associated with vitamin D deficiency [4,5,6]. For instance, a study by Nigwekar et al. (2012) showed the prevalence of vitamin D levels below 30 ng/mL to be 71% for CKD stage G3a/b, 84% for stage G4 (eGFR of 15–29 mL/min/1.73 m²), and 89% for stage G5 (eGFR < 15 mL/min/1.73 m²) [7].

**Figure 1:**
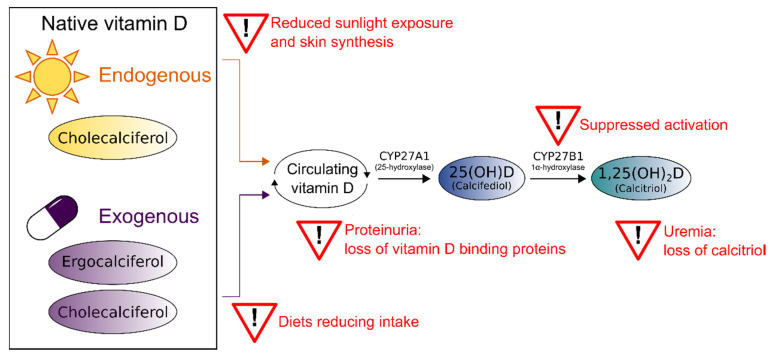
Possible reasons for vitamin D deficiency in chronic kidney disease (CKD). Adapted from Christensen et al. [10] and Nigwekar et al. [8].

For multiple reasons, CKD patients often face deficiencies in both inactive vitamin D (calcidiol or 25-hydroxyvitamin), as well as active vitamin D (calcitriol or 1,25-dihydroxyvitamin D). This might be caused by impaired skin synthesis or prescribed dietary restrictions reducing the availability of the 25-hydroxyvitamin D precursors cholecalciferol/ergocalciferol [8]. Also, CKD suppresses the 1α-hydroxylase CYP27B1, which catalyzes the activation of 25-hydroxyvitamin D [8,9]. In addition to impaired biosynthesis, CKD-associated proteinuria and uremia lead to the loss of vitamin D binding proteins and 1,25-dihydroxyvitamin D [8]. Figure 1 summarizes the causes of vitamin D loss in CKD patients. 

### 1.2. Consequences of Vitamin D Deficiency in CKD

**Figure 2:**
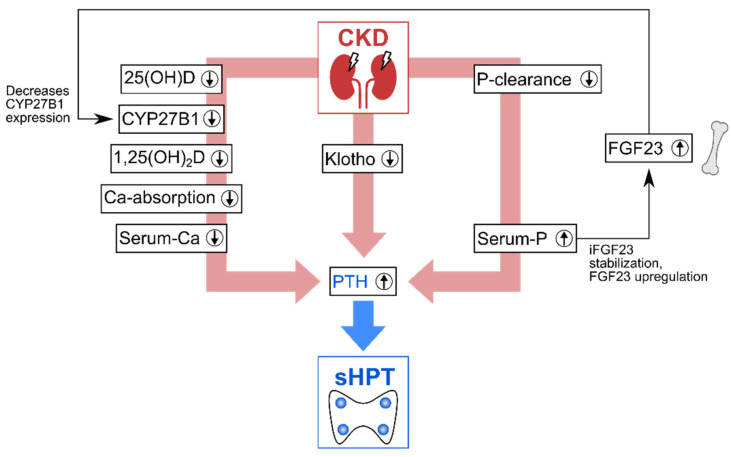
Main pathways in which chronic kidney disease (CKD) leads to elevated parathyroid hormone (PTH) levels, causing secondary hyperparathyroidism (sHPT). Circled arrows indicate increases (↑)/decreases (↓). Adapted from Germain (2020) [13] and Leifheit-Nestler and Haffner (2021) [14]. CKD—chronic kidney disease, 25(OH)D—Calcidiol, 1,25(OH)_2_D—Calcitriol, Ca—calcium, P—phosphorus, FGF23—Fibroblast growth factor-23, iFGF23—intact FGF23, PTH—parathyroid hormone, CYP27B1—1α-hydroxylase.

Vitamin D deficiency in CKD directly impacts the homeostasis of calcium and phosphate: under physiological conditions, regulatory feedback loops maintain this homeostasis, with vitamin D, the fibroblast growth factor-23 (FGF23), and the parathyroid hormone (PTH) acting as additional regulators [11,12]. To maintain calcium homeostasis, a complex of vitamin D, the vitamin D receptor (VDR) and the retinoid X receptor binds to the vitamin D response element to regulate the transcription of genes for calcium homeostasis, including epithelial calcium channels and calcium-binding proteins [10,11,12,13]. A 1,25-dihydroxyvitamin D deficiency in CKD results in insufficient induction of these genes and thus impedes the transport of active Ca^2+^ from the intestine to the circulation. The PTH from the parathyroid glands counteracts hypocalcemia by stimulating osteoclasts to initiate calcium resorption from the bone. In CKD, however, several mechanisms lead to PTH overproduction, also known as hyperparathyroidism (HPT). This type of PTH overproduction is a secondary HPT (sHPT) to differentiate it from an HPT caused by parathyroid gland disorders (primary HPT). As Figure 2 summarizes, CKD can lead to sHPT via three main pathways. 

The first main pathway that causes sHPT development is the 1,25-dihydroxyvitamin D deficiency, which results in decreased Ca^2+^-serum levels. On the one hand, 1,25-dihydroxyvitamin D decreases biosynthesis of PTH [11]. On the other hand, the decrease in Ca^2+^-serum-levels triggers PTH production. The second main pathway is the reduced renal phosphate clearance in CKD leading to hyperphosphatemia, which further stimulates the development of sHPT, for multiple reasons. For instance, phosphate directly stimulates secretion of PTH [15]. In addition, high phosphate levels also lower serum-Ca^2+^ levels by forming insoluble Ca—P complexes and by decreasing the expression of the 1α-hydroxylase CYP27B1, which further aggravates vitamin D deficiency-related hypocalcemia. Additionally, phosphate stabilizes intact FGF23 [14], enhancing the secretion of the FGF23 [16], which also downregulates the expression of CYP27B1 [17]. The third main pathway is the CKD-induced suppression of transcription of the FGF23 coreceptor Klotho: without Klotho, FGF23 cannot downregulate PTH and serum phosphate [6,11,14,15].

As these interlinked pathways to sHPT show, CKD disrupts a system of tight feedback loops, which results in an insufficient homeostasis of vitamin D and minerals.

### 1.3. Vitamin D Deficiency and Parathyroid Overproduction Lead to Mineral and Bone Disorders

The development of sHPT and the disruptions in vitamin D and mineral metabolism are part of a clinical syndrome termed CKD–mineral bone disorder (CKD–MBD) [18,19]. The clinical consequences of CKD–MBD encompass parathyroid gland hyperplasia, vascular calcification and bone abnormalities [11,20]. When CKD advances, the parathyroid glands undergo nodular hyperplasia due to constant overstimulation [20,21]. They become less sensible to vitamin D and calcium signals due to the loss of respective receptors [20]. In severe cases, this leads to patients refractory to medical treatment requiring parathyroidectomy [11]. The disruption of the mineral homeostasis in CKD–MBD increases the risk for vascular calcification, and thus also for cardiovascular diseases [11]. High serum phosphate levels causing continuous depositions of calcium phosphate salts are the reason why vascular calcification is regarded as a defining characteristic of CKD–MBD, greatly increasing mortality [22,23]: a study by Górriz et al. identified cardiovascular events as the leading cause of death (36%) in non-dialysis CKD patients [24]. The eponymous bone disorders in CKD–MBD are a consequence of the hypocalcemia and PTH overproduction inducing excessive bone resorption through osteoclast stimulation [11,17]. The term renal osteodystrophy encompasses the different patterns in which CKD–MBD impairs bone quality and quantity, with osteistis fibrosa and adynamic bone disease regarded as the main conditions [25]. The bone abnormalities lead to osteoporosis [26] and the incidence of fractures increases with CKD progression [27]. Overall, vitamin D deficiency and sHPT result in a complex pathophysiology, making their control in patients with CKD and on dialysis challenging.

### 1.4. The Ongoing Challenge of Optimal Vitamin D Supplementation in Renal Hyperparathyroidism

For more than 40 years, vitamin D deficiency and the endocrine reaction with consecutive PTH overproduction has been taught to nephrology residents already early in their training. Insufficient vitamin D metabolism is regarded as a fundamental step in the development of renal HPT, which in turn is a dominant clinical issue in CKD, as well as dialysis patients. Accordingly, studies on CKD patients show that serum PTH is an independent predictor for vascular death, fractures, and mortality [28] and that higher PTH levels are associated with a higher cost of care [29].

Looking back at the long history of our understanding of vitamin D deficiency in CKD, it is interesting to detect a significant evolution in our attitude towards the optimal therapy and treatment goals over time. Accordingly, nephrology has experienced quite substantial modifications over time in how we have performed vitamin D replenishment in non-dialysis-CKD patients and in patients on dialysis. The following review intends to summarize these evolutionary changes following the different treatment approaches and outlines their rationale, mechanisms, and treatment and side effects to us. Each new approach taught us lessons important for the next evolutionary step in up-to-date therapy of vitamin D deficiency and optimal treatment of renal HPT.

## 2. Active Vitamin D Substitution and the Unwanted Side Effects of Overtreatment

The publication of the first guidelines on diagnosis and treatments of CKD–MBD patients by the Kidney Disease: Improving Global Outcomes (KDIGO) initiative in 2009 underlined the importance of addressing CKD–MBD [30]. At that time, nephrology already had learned to use calcitriol with more restraint, after discovering the associations between hypercalcemia, hyperphosphatemia, vascular calcification, and adynamic bone disease. Based on the rationale and background data described below, these KDIGO guidelines defined clear limits to avoid overtreatment with (active) vitamin D (including vitamin D analogues). These limits were PTH oversuppression and the development of hypercalcemia and hyperphosphatemia [30].

### 2.1. Active Vitamin D Substitution as the Beginning of Renal HPT Treatment

Clinicians already investigated decades ago if supplementation with active vitamin D or one of its precursors suffices to treat vitamin D deficiency in CKD/dialysis patients with sHPT. In the 1970s and early 1980s, several small, randomized studies in CKD or dialysis patients detected improvements regarding the PTH-level. For instance, Memmos et al. reported that in dialysis patients, oral supplementation of calcitriol successfully arrested or reversed sHPT in patients with a mean PTH of 205 µEq/mL, while the response in those with higher PTH-levels (mean of 709 mEq/mL) was slower [31]. The PTH reduction in the latter to 445 µEq/mL was insufficient, so the authors concluded that parathyroidectomy should be preferred in advanced HPT unless the treatment response is fast [31]. The results of Sharman et al. [32] supported the effects of PTH-suppression by active vitamin D, concluding that this treatment might prevent or restrain bone disorders. In contrast, hopes for immediate-release calcidiol were shattered early on in the 1970s, as studies could not detect reliable PTH reductions with this treatment [33,34]. For native vitamin D (cholecalciferol, ergocalciferol), studies have also failed to provide evidence for reliable, effective PTH control over the years [35,36,37]. Hence, calcitriol substitution emerged as the standard of care in renal HPT early on [38]. 

### 2.2. Active Vitamin D-Induced Hypercalcemia, Hyperphosphatemia and Vascular Calcification

Some of the early studies on active vitamin D usage already detected hypercalcemia as a potential unwanted side effect [39,40], which later turned out to be a core concern of this treatment. Given that calcitriol mediates the intake of calcium and phosphorus, it appears obvious that overtreatment with calcitriol causes hypercalcemia and hyperphosphatemia [41,42,43,44]. These high serum levels result in systemic effects: a study by Goodman et al. (2000) highlights vascular calcification as a major adverse effect and exemplifies how around the millennium change, awareness for the potential harms of active vitamin D replenishment was rising [45].

Goodman et al. investigated 39 young hemodialysis patients with electron-beam CT scanning. Although the authors detected no coronary artery calcification in patients younger than 20 years, they found it in 14 of 16 patients 20–30 years of age. Compared to patients without coronary artery calcification, those with calcification were older (26 ± 3 vs. 15 ± 5 years, *p* < 0.001) and had been on dialysis treatment for a longer period. Also, patients with coronary artery calcification had higher mean values for serum phosphorus levels, calcium–phosphorus ion product in serum, and the daily calcium intake. Follow-up CT scanning of 10 patients with calcification showed that the calcification score substantially increased from 125 ± 104 to 249 ± 216 (*p* = 0.020) over a mean period of almost 2 years. These impressive findings contributed to a large extent to a substantial shift in nephrologists’ attitude towards (active) vitamin D replenishment in order to treat renal HPT. 

While the association between end-stage renal disease and cardiovascular disease had been known for ages at that time [46], it was the above-mentioned Goodman et al. paper that systematically quantified the magnitude of the problem. Even more importantly, this publication conveyed the issue of cardiovascular disease in general and cardiovascular calcification in particular to a broader audience. Soon, elevated calcium levels and high calcium–phosphate product levels were identified and generally accepted as potential risk factors for the development of cardiovascular disease in CKD patients [47,48]. Since the application of (high dosage) vitamin D (especially in the active form calcitriol) associates virtually inevitably with the development of hypercalcemia and hyperphosphatemia in CKD patients [49], the uncontrolled dosing of calcitriol against renal HPT eventually received a bad reputation.

However, the aetiology of vascular calcification is more complex. For sure, excessive supplies of vitamin D, calcium, and phosphate are required for providing a pro-calcific physicochemical environment and hypercalcemia and hyperphosphatemia are accepted potent driving forces of vascular calcification in patients with renal disease [50]. However, vascular calcification also requires an imbalance between pro-calcific substrates and calcification inhibitors/inducers, e.g., fetuin-A [51], pyrophosphates (inhibitors) or the transforming growth factor-β1 (inducer) [50,52].

### 2.3. Active Vitamin D-Induced Adynamic Bone Disease

In parallel to the increasing awareness that too much active vitamin D might exaggerate calcium and phosphate metabolism disturbances, more and more data indicated the potential association between active vitamin D overtreatment and the development of adynamic bone disease [53]. Adynamic bone disease is a condition with substantially reduced cellular activity in bone. As a consequence, the calcium and phosphate buffering capacity of the bone compartment is diminished, and as such adynamic bone disease drives these elements towards extraosseous deposition [54]. Hence, it became clear that the association between vitamin D treatment and the outcome in CKD patients follows a U-shaped dose–response curve [55]. There are relevant clinical consequences to be expected on the side of vitamin D undersupply (progressive renal osteodystrophy, bone demineralization and uncontrolled HPT), however, there is also a threat of oversupply by filling the body with too much calcium and phosphate and preventing their physiological deposition in the bone compartment. Taken together, nephrologists’ attitude towards vitamin D treatment changed substantially and the time of liberal (active) vitamin D treatment ended around the millennium change. Moreover, studies indicated a clear association between calcitriol therapy and the increasing levels of FGF23 in renal patients [56]. Even if direct cardiotoxic effects of FGF23, e.g., inducing left ventricular hypertrophy, are still a matter of debate, this upregulation in FGF23 levels is a reason for concern, since high levels of FGF23 are strongly and independently associated with reduced survival and increased cardiovascular risk in renal patients both before and on dialysis [57,58].

## 3. Alternative Vitamin D Substances and Efforts to Avoid Calcium Overload

In parallel to the perception that overt and even latent hypercalcemia were noxious in patients with CKD or on dialysis, clinical investigations for calcium-free phosphate binders intensified [59]. Whereas some trials investigating calcium-free phosphate binders with surrogate endpoints, such as vascular calcification progression, revealed promising results [60], the first large randomized interventional trial with hard clinical endpoints failed to demonstrate an improvement in survival rate with a calcium-free phosphate binder (DCOR trial) [59]. Hence, nephrologists’ hopes were placed in alternative vitamin D formulations with the potential to effectively treat renal HPT but with less tendency to induce hypercalcemia. Novel vitamin D analogues, such as maxacalcitol, doxercalciferol or paricalcitol, came into play, with the latter predominantly in use in Europe and the US. It was claimed that these analogues have all the good properties of vitamin D and showed less unwanted side effects, especially less tendency for hypercalcemia. As vitamin D analogues, the mechanism of action for these substances was basically the same as for 1,25-hydroxyvitamin D, namely binding to the VDR to mediate PTH lowering. However, it was conceived that side-chain modifications would alter the binding affinity to circulating vitamin D binding proteins and/or the VDR to such a degree that this would allow for better control of the biochemical (side) effects [61].

**Figure 3:**
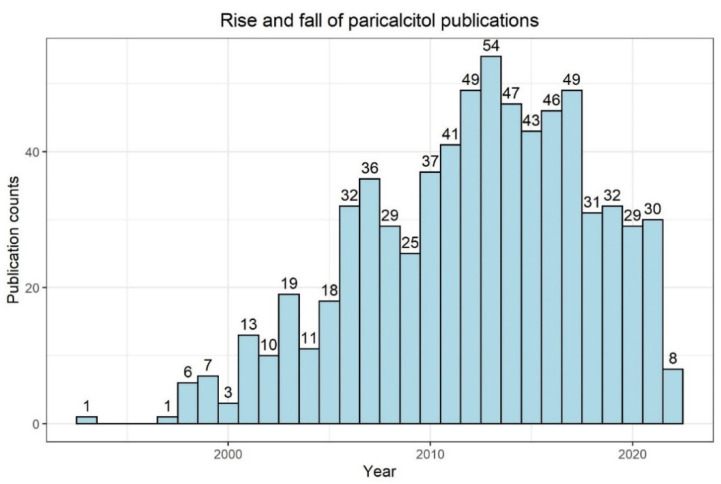
PubMed search results for “paricalcitol” (as of 24 May 2022; https://pubmed.ncbi.nlm.nih.gov/, accessed on 24 May 2022). The bin width is 1 year. Numbers on top of bars indicate counts.

Early studies in humans showed the efficacy of paricalcitol in terms of PTH suppression in hemodialysis patients with HPT [62]. It is noteworthy that an interventional study reported that paricalcitol effectively lowered intact PTH serum levels in hemodialysis while having a safety profile comparable to placebo [63]. Later, the nephrology community evaluated with great interest human (non-interventional) data associating the usage of paricalcitol with better outcomes and better survival compared to calcitriol [64,65]. However, well-designed randomized, prospective, interventional trials evaluating cardiovascular surrogate (intermediate) endpoints failed to show significant benefits of paricalcitol [66]. Moreover, growing clinical experience, as well as study data underlined the fact that even the novel vitamin D analogues are not neutral in terms of risk for hypercalcemia development [41]. According to a PubMed search in May 2022, the number of annual publications with “paricalcitol” has been decreasing for several years (Figure 3), possibly indicating a consecutive decrease in clinical and scientific interest in the substance due to the lack of reliable superiority compared to calcitriol.

It is noteworthy that none of these novel vitamin D analogues underwent high quality randomized, prospective testing in humans with studies evaluating relevant hard endpoints (such as myocardial infarction, stroke, or survival). This lack of evidence fits well in today’s disappointing picture that nephrology in general shows a remarkable lack of successful evidence-creating interventional trials providing clear and significant results with the power to modify state-of-the-art treatment. This issue is especially true for HPT treatment.

## 4. Extended-Release Calcifediol: A Combination of Efficacy and Safety as the Next Step in sHPT Treatment

A novel form of vitamin D holds promise in several respects: extended-release calcifediol (ERC; EU term: prolonged-release calcifediol) is an orally administered prohormone of 1,25-dihydroxyvitamin D. The area of indication is secondary HPT in CKD stage G3–G4 patients. The key difference to other vitamin D supplements is the formulation: the calcifediol is encapsulated in a lipophilic, wax-like structure that allows prolonged calcifediol release over an extended 12 h period [42,67]. This has important biochemical implications: By avoiding the rapid rises of 25-hydroxyvitamin D and 1,25 dihydroxyvitamin D levels, as seen with immediate-release calcifediol, ERC does not stimulate the negative feedback loop leading to increased activity of the enzyme CYP24A1, which degrades active vitamin D to inactive forms [67]. Two phase 3 clinical trials investigated the efficacy and safety of oral ERC in patients with CKD stage G3–G4 [42,68]. In these studies, 429 patients with CKD stage G3–G4, secondary HPT and vitamin D insufficiency were treated with 30 μg ERC or placebo daily for 12 weeks, 30 or 60 μg ERC or placebo for 14 weeks, then 30 or 60 μg ERC for up to 52 weeks (extension study). These data show that ERC application steadily increased serum 25-hydroxyvitamin D levels, with ≥80% of patients reaching levels of at least 30 ng/mL (placebo: ≤7%). The primary endpoint was defined as ≥30% reduction in PTH from baseline at week 26. The endpoint was achieved by 33% and 34% of patients in each study in the ERC group (versus 8% and 7%, respectively, with the placebo). In the open-label extension phase of the trial, patients primarily treated by placebo, and then switched to ERC experienced a comparable decline in plasma PTH levels compared to those patients initially actively treated with ERC in the blinded study phases. Hence, ERC proofed efficacy in renal HPT treatment. The CKD stage did not influence the level of PTH suppression induced by specific total 25-hydroxyvitamin D levels.

As already discussed, the efficacy of vitamin D treatment measured by PTH reduction in secondary HPT is not the entire story. What kind of treatment-emergent adverse events were recorded? Do we again achieve effective lowering of sHPT at the expense of safety issues related to calcium and phosphate levels? The data for ERC look promising: hyperphosphatemia and hypercalcemia occurred to a comparable extent in both the ERC treatment and placebo of the phase 3 trials. There were minimal changes in serum calcium and phosphate, and hence a low risk for hypercalcemia and hyperphosphatemia was recorded. The gradual elevation of 25-hydroxyvitamin D with ERC to levels as high as 92.5 ng/mL over a 26-week period had no adverse effects on safety parameters [42,68]. These trial data are supported by emerging real-world data by Fadda et al. (2021) [69]. This study confirmed ERC’s effectiveness in increasing serum 25-hydroxyvitamin D and reducing PTH levels without a statistically significant or notable impact on serum calcium and phosphate levels (on average, calcium went from 9.2 ± 0.1 mg/dL to 9.3 ± 0.1 mg/dL and phosphate went from 3.8 ± 0.1 mg/dL to 3.9 ± 0.1 mg/dL). Of 174 patients prescribed ERC, 70.1% (122 patients) achieved 25-hydroxyvitamin D-levels of ≥30 ng/mL and 40.2% of patients achieved a PTH reduction of ≥ 30%. In summary, these data indicate that on a biochemical level, ERC is a true step forward in HPT treatment.

## 5. Will the Usage of SGLT2-Inhibitors Facilitate CKD–MBD Research?

Regarding vitamin D deficiency and renal HPT treatment, there remain some major gaps in the evidence even if preclinical and clinical research have now been conducted intensively for more than 40 years. We still lack clear and substantiated evidence which form of vitamin D supplement to use in order to titrate HPT and which specific PTH level we should aim at. Accordingly, the KDIGO CKD–MBD guidelines lack recommendations on target PTH levels, as well as treatment algorithms for vitamin D replenishment in renal HPT [30]. The latter issue is of outstanding importance regarding the fact that the PTH level and outcome have a U-shaped relationship [70]. 

While HPT treatment in particular and CKD–MBD treatment in general somehow stagnate without providing novel breakthrough study results, the nephrology community found some novel and promising treatment options outside CKD–MBD with the potential to be real game changers. High-quality RCTs in recent years showed that true outcome improvement and retardation of kidney disease progression are indeed compatible with each other: The SGLT2-inhibitors and the non-steroidal mineralocorticoid receptor blocker finerenone reduce both major cardiovascular events (MACE) and major renal events (“MAREs”), such as renal death and the initiation of dialysis or renal transplantation [71,72]. Maybe this positive experience could stimulate researchers and industry to start comparable trials for non-dialysis CKD–MBD patients to provide clear guidance regarding the optimal starting point, dosage, and type of future vitamin D treatment, as well as phosphate management.

In addition to that, the safety, efficacy and widespread usage of these novel drugs in CKD treatment might also stimulate further research in renal HPT, vitamin D, and CKD–MBD: Why that? Interestingly, the above-mentioned SGLT2-inhibitors promote the development of hyperparathyroidism and changes in other markers of CKD–MBD. Small prospective trials have shown that the usage of SGLT2-inhibitors influence CKD–MBD parameters. An RCT by de Jong et al. (2019) tested dapagliflozin in 31 patients with diabetic kidney disease [73]. This study showed that compared with placebo, dapagliflozin increased serum phosphate by 9%, PTH by 16%, FGF23 by 19%, and decreased serum 1,25-dihydroxyvitamin D by 12%. Calcium and 25-hydroxyvitamin D were unaffected [73]. Similar changes occurred in diabetic patients treated with empaglifozin [74]; compared to the baseline, 3 days of empagliflozin treatment significantly increased serum levels of phosphate (from 1.10 ± 0.21 mmol/L to 1.25 ± 0.23 mmol/L), PTH (from 57.40 ± 30.49 pg/mL to 70.23 ± 39.25 pg/mL), and FGF23 (from 77.92 ± 24.31 pg/mL to 109.18 ± 58.20 pg/mL) and decreased 1,25-dihydroxyvitamin D (from 35.01 ± 14.01 ng/L to 22.09 ± 10.02 mg/L). This biochemical constellation points towards hyperphosphatemia development as the initial step triggering a cascade of changes in CKD–MBD parameters: the authors hypothesize that the inhibition of the SGLT2 receptor leads to increased sodium concentration in the lumen, which in turn leads to an increased phosphate re-absorption in the proximal tube by NaPi cotransporters. Assumingly, this re-absorption of phosphate into the blood causes the temporal increase in serum phosphate, triggering an increase in FGF23 and PTH, which counteract elevated phosphate levels. Importantly, these changes were transient and no differences of these parameters were recorded after 3 months of treatment. It is unclear if there is an important clinical meaning in the findings and if preventive therapeutic action is indicated. In summary, there is apparently a substantial period of time in which SGLT2-inhibitors induce a mean, potentially harmful change in CKD–MBD parameters. Maybe, hyperphosphatemia, hypovitaminosis D and HPT secondary to SGLT2-inhibitor applications mask at least transiently some of the overall beneficial effects upon MACE and MARE. Further long-term research is necessary to investigate if these transient changes in CKD–MBD parameters modify the overall beneficial outcome improvement of the SGLT2-inhibitors in patients with diabetes, CKD, and heart failure. Comparable effects of MRAs have not been documented: To the best of our knowledge, it has not been evaluated if finerenone modifies PTH levels in specific patient groups. There was no significant influence of eplerenone therapy upon PTH levels in patients with primary HPT [75]. To conclude, it should be noted that the higher survival of CKD stage G2/G3a patients due to SLGT2-inhibitors and finerenone might additionally enhance the interest in CKD–MBD research: after all, CKD–MBD is primarily an issue in later CKD-stages.

## 6. Vitamin D Treatment in Summary: A Call for Action

**Figure 4:**
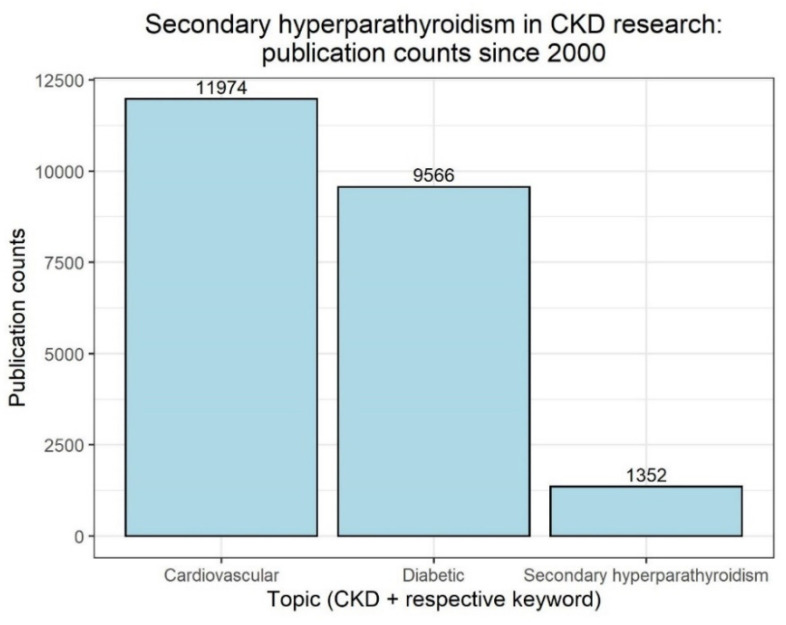
PubMed search results from 2000 to 2022 for “CKD” and one of the following keywords, as of 25 May 2022: “Cardiovascular”, “Diabetic” or “Secondary hyperparathyroidism”; https://pubmed.ncbi.nlm.nih.gov/, accessed on 25 May 2022). Numbers on top of bars indicate counts.

Facing the convincing data about the possibility of outcome improvement in renal patients outside CKD–MBD mentioned above, we indeed speculate about a potentially changing focus in treatment of CKD patients. We anticipate the threat of a future neglect to sHPT: sHPT and vitamin D deficiency treatment might fall into oblivion compared to novel treatment regimens providing evidence-based options to improve renal and cardiovascular outcomes and to reduce mortality. A look back at the publication numbers of the last 20 years further underlines this point, as in the context of CKD, publications with cardiovascular or diabetic topics far outnumber those with sHPT (Figure 4).

**Figure 5:**
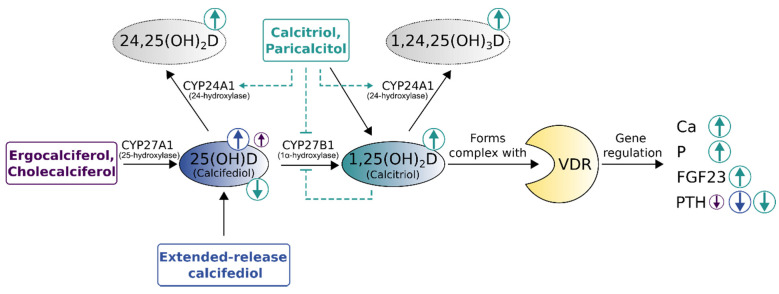
Comparison of vitamin D replenishment treatments for non-dialysis chronic kidney disease patients with secondary hyperparathyroidism. Size and direction of circled arrows indicate the magnitude and effect (increase [↑]/decrease [↓]) of treatments on laboratory parameters. The immediate surge in vitamin D-levels by calcitriol bolus supplementation carries the risk of triggering vitamin D catabolism (dashed lines). VDR—vitamin D receptor, 24,25(OH)2D—24,25-dihydroxyvitamin D (inactive prohormone), 1,24,25(OH)3D—1,24,25 trihydroxyvitamin D (inactive hormone), Ca—calcium, P—phosphorus, FGF23—Fibroblast growth factor-23, PTH—parathyroid hormone, CYP27A1—25-hydroxylase, CYP27B1—1α-hydroxylase, CYP24A1—24-hydroxylase. Adapted from Cozzolino et al. [41], Christensen et al. [10], extended with data from Sprague et al. [42,68] and Fadda et al. [69].

Where does nephrology go from here? The authors clearly underline that the topic sHPT in renal disease is not dead. The novel 2017 KDIGO CKD–MBD guidelines recommend regular monitoring of PTH levels in patients starting on CKD stage G3a. This monitoring is intended to identify patients with elevated or rising PTH levels, so that these patients at risk can be evaluated for modifiable risk factors and the need for specific interventions [76]. Among these potential interventions, the guidelines specifically name measuring vitamin D levels in CKD patients with HPT in order to detect this modifiable risk factor [76]. With our experience from the past and novel treatment options (such as ERC), we have the potential to treat renal HPT in a safe and effective way in the future. Extended-release calcifediol may help us to transfer the well-known efficacy of calcitriol into a modern HPT treatment with less risk for hypercalcemia and hyperphosphatemia induction (Figure 5). 

However, renal HPT treatment has to hold its own against other forms of renal treatment, such as SGLT2-inhibition or finerenone. Those substances have similarly shown beneficial influences on renal surrogates (slowdown of eGFR decline, reduction in proteinuria, and others) just as ERC did in PTH reduction in patients with sHPT. Nonetheless, ERC requires studies proving clinical meaningful influence also upon hard endpoints, including mortality. A first notable step has been done by ERC in fulfilling our needs for a safe and effective modification of biochemistry in CKD–MBD. The final high-grade evidence about the role of ERC in modifying hard clinical endpoints is pending. In terms of HPT treatment, such hard endpoints could be cardiovascular (“MACE”) and also extra-cardiovascular events, such as fracture incidence or the need for parathyroidectomy. With the experience of more than 40 years of HPT treatment and with modern vitamin D formulations, such as ERC, in our hands, it is now time for the nephrology community to work towards the solution for three core HPT issues:

1. What is the optimal PTH target level for CKD and dialysis patients?
2. What is the optimal vitamin D level to support optimal PTH titration?
3. How can HPT treatment support reduction in the occurrence of hard renal and cardiovascular events in CKD and dialysis patients?

## 7. Future Directions

The core issues in renal HPT presented in this review are all interlinked: on the one hand, the lack of CKD-specific reference ranges/targets for PTH and vitamin D complicates the decision making for which levels or developments of PTH are alarming for CKD patients. On the other hand, both the lack of comparative studies and renal or cardiovascular outcome data (especially for ERC) prevents the development of treatment algorithms. Solving these core issues might finally pave the way to a clear guideline-recommended treatment algorithm in renal HPT.

Systematic analyses of epidemiological studies might facilitate determining putative PTH and vitamin D references ranges. In terms of real-world data, for example, a prospective study by Isakova et al. (2020) [4] reports the dynamics of vitamin D, PTH, and mineral markers for 847 CKD patients progressing to ESRD over a span of 8 years; as mean eGFR declined from 32.2 to 10.1 mL/min/1.73 m^2^, median PTH-levels steadily increased to more than twice the baseline value, from 74.1 to 156.5 pg/mL. The authors also describe how abnormalities of mineral metabolism intensified 3 years into the study (5 years prior to ESRD) and how the percentages of active vitamin D and nutritional vitamin D prescriptions increased. It would be interesting to compare how the dynamics change according to the type of vitamin D treatments in a real-world setting, including ERC.

However, laboratory parameters are one side of the coin, clinical endpoints are the other. Comparative studies with the different vitamin D replenishment strategies should, therefore, also analyze suitable renal and cardiovascular endpoints. For calcitriol, Selamet et al. (2018) showed that in geriatric patients, lower calcitriol levels were an independent risk factor for >30% eGFR decline (mean observation time 6.4 years) [77]. Also, the effects of HPT-treatment on FGF23 and phosphate homeostasis should be taken into account: RCTs with dialysis patients have shown that strict phosphate control significantly delays progression of coronary artery calcification [78] and that FGF23 suppression with the calcimimetic etelcalcetide inhibits left ventricular hypertrophy [79].

## 8. Conclusions

Vitamin D replenishment remains a core issue in the treatment of sHPT in CKD patients, in particular the risk of the side effects of hypercalcemia and hyperphosphatemia. In light of the advances made in diabetic nephropathy and cardiovascular outcomes for CKD patients, nephrology needs to catch up. Long overdue is the resolution of the core issues in renal HPT, the establishment of clear and substantiated evidence on the optimal choice of vitamin D treatment, target levels for vitamin D and PTH, and how HPT treatment ameliorates renal and cardiovascular clinical outcomes. With developing treatment options, such as ERC, treatment of vitamin D deficiency and sHPT in CKD patients might leap into its next phase after more than 40 years. 

## Data Availability

Not applicable.
